# Novel Perspectives Regarding the Pathology, Inflammation, and Biomarkers of Acute Respiratory Distress Syndrome

**DOI:** 10.3390/ijms22010205

**Published:** 2020-12-28

**Authors:** Pradeesh Sivapalan, Barbara Bonnesen, Jens-Ulrik Jensen

**Affiliations:** Respiratory Medicine Section, Department of Internal Medicine, Herlev and Gentofte Hospital, University of Copenhagen, 2900 Hellerup, Denmark; BBBB@dadlnet.dk (B.B.); jens.ulrik.jensen@regionh.dk (J.-U.J.)

**Keywords:** acute respiratory distress syndrome, biomarkers, inflammation, molecular pathway, therapeutics

## Abstract

Acute respiratory distress syndrome (ARDS) is an acute inflammation of the lung resulting from damage to the alveolar–capillary membrane, and it is diagnosed using a combination of clinical and physiological variables. ARDS develops in approximately 10% of hospitalised patients with pneumonia and has a mortality rate of approximately 40%. Recent research has identified several biomarkers associated with ARDS pathophysiology, and these may be useful for diagnosing and monitoring ARDS. They may also highlight potential therapeutic targets. This review summarises our current understanding of those clinical biomarkers: (1) biomarkers of alveolar and bronchiolar injury, (2) biomarkers of endothelial damage and coagulation, and (3) biomarkers for treatment responses.

## 1. Introduction

Acute respiratory distress syndrome (ARDS) is characterised by uncontrolled inflammation and damage to endothelial and epithelial barriers of the lung. It results in increased permeability of the alveolar–capillary membrane, infiltration of inflammatory cells, and excessive release of cytokines and chemokines, and it leads to acute noncardiogenic pulmonary oedema. Clinical symptoms associated with ARDS include severe hypoxia, difficulties performing gas exchange, impairment of lung mechanics, and respiratory failure [1,2]. A panel of experts assembled in 2011 (an initiative of the European Society of Intensive Care Medicine endorsed by the American Thoracic Society and the Society of Critical Care Medicine) and developed the Berlin Definition of ARDS using a consensus process. The Berlin definition requires all four criteria depicted in Table 1 to be present for a diagnosis of ARDS (Table 1) [3].

ARDS is associated with several risk factors, including pulmonary and non-pulmonary sepsis, severe pneumonia, pulmonary contusions, trauma, drug overdose, and aspiration of gastric contents [2]. Despite recent progress, it remains difficult to successfully treat sepsis and ARDS, and because the underlying molecular mechanisms are not completely understood, ARDS mortality rates remain unacceptably high [4].

Biomarkers may be useful for identifying ARDS, stratifying risks, and predicting specific outcomes (e.g., mortality). They may also be used for assessing the severity of illnesses, revealing prognoses, and monitoring responses to therapy. Therefore, diagnostic biomarkers of ARDS may be used to identify those patients who are most likely to benefit from therapeutic interventions. Although several ARDS biomarkers have been identified, none of them are considered reliable enough for clinical application. It is unlikely that any single biomarker will be able to predict the risk of ARDS, diagnose the disease, or determine prognoses with complete accuracy. However, there may be particular sets of markers that can be used to identify groups of patients with particular characteristics associated with the severity of illness and prognosis (Table 2).

**Figure 1:**
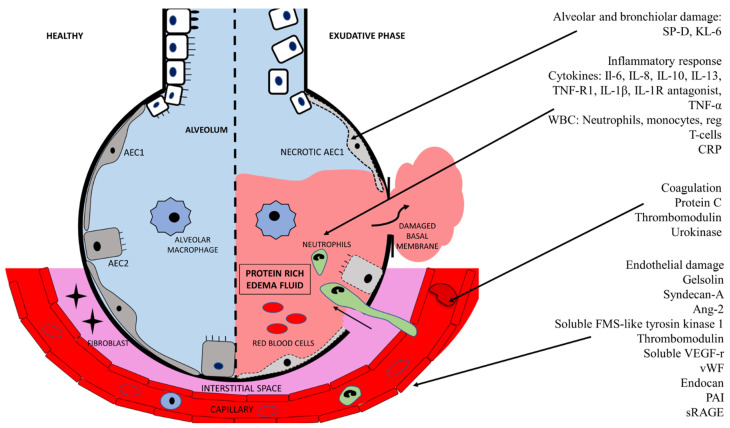
Biomarkers of acute respiratory distress syndrome organised in alveolar/bronchiolar damage, inflammatory response, coagulation, and endothelial damage.

In this review, we describe some of the most promising ARDS biomarkers. We focus on three areas of interest: (1) alveolar and bronchiolar injury, (2) endothelial damage and coagulation, and (3) biomarkers for treatment responses (Figure 1).

## 2. Alveolar and Bronchiolar Injury Biomarkers

### 2.1. Surfactant Protein D

Surfactant protein D (SP-D) is mainly produced by alveolar type II cells in the lung epithelium, and it is secreted to the surfactant layer of the alveoli. SP-D expression is stimulated by lung injury or infection and may then be detected in plasma. Plasma SP-D levels have been used as a biomarker for lung injury, particularly alveolar epithelial injury [5], and SP-D levels are higher in patients with more severe lung injuries and those who have more severe outcomes, including prolonged mechanical ventilation and death [6,7,8]. SP-D predicted mortality in patients with ARDS (*N* = 528) in a cohort-based on the ALVEOLI Randomised Controlled Trial (RCT) [8]. In a cohort based on two RCTs (*N* = 547 and *N*= 500), a combination of three biomarkers (interleukin (IL)-8, soluble tumour necrosis factor receptor-1, and SP-D) had prognostic value on mortality [9]. A mortality prediction model for ARDS that included age, APACHE III, SP-D, and IL-8 performed well in a validation cohort with 849 patients in the National Heart, Lung, and Blood Institute ARDSNet Fluid and Catheter Treatment Trial (FACTT), 144 patients from a clinical trial of sivelestat for ARDS (STRIVE), and 545 ARDS patients from the VALID observational cohort study [10]. SP-D levels can be used to identify patients with alveolar lung disease and to stratify these patients according to risk. They also correlate with the severity of the illness. In addition, plasma SP-D levels can be helpful to diagnose ARDS [11], because patients with ARDS exhibit an increase in SP-D levels, which peaks between days 3 and 7 of the illness [6]. This has been shown in several studies, e.g., high levels of SP-D within 48 h after intensive care unit (ICU) admission (*N* = 407) might serve as a diagnostic marker for ARDS in patients hospitalised in medical ICU [11] and patients with severe sepsis (*N* =100) [12]. In summary, plasma SP-D appears to be a promising biomarker in ARDS.

### 2.2. Krebs von den Lungen-6

A second biomarker that is released by injured Type II pneumocytes is glycoprotein Krebs von den Lungen-6 (KL-6), also known as MUC1. KL-6 was initially associated with non-Small Cell Lung Cancer, and a high initial KL-6 level in serum or plasma may predict poor clinical outcome, including 5-year survival in these patients [13,14,15,16]. KL-6 has also been associated with interstitial lung disease and may be elevated, especially during exacerbation in these patients [17], and KL-6 has been linked to obstructive sleep apnea, where it has been proposed to reflect the degree of subclinical lung injury associated with obstructive sleep apnea [18,19]. Finally, KL-6 has been analysed in small case-control studies of patients with ARDS. An increase in KL-6 levels may also indicate alveolar injury, and although KL-6 is a nonspecific marker, patients with ARDS show increased levels of KL-6 in the bronchoalveolar lavage fluid and plasma [20]. One study showed that levels of KL-6 had increased significantly in the epithelial lining fluid of patients with ARDS who later died. Additionally, an increase in KL-6 levels was detectable from disease onset, suggesting that KL-6 levels may be used to determine prognoses [20]. KL-6 might be elevated; it possibly increases over time as the disease progresses, and it possibly to some extent reflects the subtype of ARDS [7,21,22]. Hence, the elevated levels of KL-6 in plasma/serum in patients with pulmonary disease does not seem capable of distinguishing between the underlying condition, and its usefulness in ARDS is unclear with the currently available data.

### 2.3. Soluble Receptor for Advanced Glycation end Products

Soluble receptor for advanced glycation end products (sRAGE) is a marker of lung epithelial injury. It is a decoy receptor that competitively inhibits signaling through membrane bound sRAGE, hereby inhibiting inflammatory cytokines such as TNF-α. Plasma levels of sRAGE could not predict ARDS in 129 patients with severe sepsis [23], nor in 230 critically ill patients [24]; however, higher levels of sRAGE predicted ARDS in a multicentre, prospective observational cohort study of 464 critically ill patients [25], and sRAGE was associated with mortality in a meta-analysis of 746 patients with ARDS based on eight trials; one large RCT-based cohort, four non-RCT based cohorts, and three case-control studies [26]. This meta-analysis was probably dominated somewhat by the RCT-based cohort of 676 patients [27], as the other cohort studies only included 21–119 patients [28,29,30,31] and the case-control studies only included 16–33 cases [7,32,33]; however, the authors do not specify their method of patient inclusion. The RCT cohort was from an RCT on mechanical ventilation strategies for patients with ARDS which was performed by the Acute Respiratory Distress Syndrome Network [34]. A combination of sRAGE and Angiopoietin-2 (Ang-2) were superior to clinical diagnosis for the diagnosis of ARDS in severe trauma [35]. In severe sepsis, a combination containing sRAGE, SP-D, and Club Cell Protein 16 was useful for the diagnosis of ARDS [12]. Therefore, in combination with other markers, sRAGE appears to be a useful diagnostic biomarker.

## 3. Endothelial Injury and Coagulation Biomarkers

### 3.1. Gelsolin

ARDS is characterised by endothelial cell damage, hypercoagulability, and intravascular fibrin deposition due to impaired fibrinolysis. The actin-scavenging protein gelsolin is continuously produced by muscle cells and serves as a physiological buffer of actin in the blood [36]. Actin is released into the blood following tissue damage. In response, gelsolin levels decrease due to the formation and subsequent clearance of actin–gelsolin complexes. Plasma gelsolin levels decrease when lung tissue is damaged, and a recent study of 700 patients who were critically ill showed that a low plasma gelsolin level was a strong predictor of poor respiratory outcomes, but not general outcomes, in mechanically ventilated patients. Therefore, gelsolin levels could be used together with SP-D levels to predict respiratory outcomes [37]. This observation requires further validation in other cohorts of critically ill ventilated patients. Although non-specific markers of endothelial injury such as soluble thrombomodulin (TM) and syndecan-A do not predict ARDS, they do predict overall prognoses for both children and adults with pre-existing respiratory failure. The ProCESS RCT (*N* = 1341) recorded baseline plasma levels for several proteins linked to endothelial cell permeability and haemostasis. This trial reported that the baseline values of several endothelial injury markers were higher in patients who died than in patients who survived. Among these markers were Ang-2, soluble fms-like tyrosine kinase 1, the soluble vascular endothelial growth factor receptor, TM, and von Willebrand factor [38].

### 3.2. Thrombomodulin

The membrane-bound endothelial cell glycoprotein TM, also known as CD141 or BDCA-3 is an important physiological anticoagulant in pulmonary capillary vessels. It may be released from the cell membrane into circulation during inflammation of almost any kind including smoking, ionising radiation, radiation pneumonitis, exacerbations of idiopathic pulmonary fibrosis, disseminated intravascular coagulation, and surgery.

Although TM does not seem to predict ARDS, TM levels may predict severity and mortality, as shown in large studies where TM was able to predict 60-day mortality in a cohort of 449 patients with ARDS based on the FACTT RCT [39], and in a likewise large study (*N* = 1103), TM was a strong and independent predictor of organ failure and 90-day mortality from all causes in patients with sepsis [40]. In a small cohort study (*N* = 75) from the APC RCT, TM predicted the severity of ARDS [41], but in a few small studies, TM could not predict mortality [41,42], which was probably due to small sample sizes (*N* = 75 and *N* = 50).

### 3.3. Protein C

TM activates Protein C (in its activated form, it is also known as Drotrecogin alfa and Autoprothrombin II-A), which seems to exert a protective function on endothelial cells. Protein C levels are also elevated in cases of pulmonary inflammation; however, only a few studies have analysed its possible role as a biomarker for ARDS. Protein C predicted 28-day mortality in both a cohort study based on the PROWESS trial (a placebo-controlled, double-blind, RCT) with 840 patients with sepsis [43] and in a cohort of 440 patients with sepsis and an average PaO_2_/FiO_2_ ratio consistent with ARDS [44]. The predictive quality of Protein C could not be verified in smaller studies [42,45], which was probably due to small sample size (*N* = 50 and *N* = 53). Hence, the role of Protein C as a biomarker looks promising with the currently available data.

### 3.4. Endocan

Endocan, also known as endothelial cell-specific molecule 1, is a proteoglycan expressed by pulmonary endothelium, which may weaken inflammatory responses by inhibiting leukocyte recruitment. Endocan has only been analysed in small studies of lung injury, and its potential as a biomarker remains unclear with the currently available evidence [45,46,47,48,49,50,51,52].

### 3.5. Plasminogen Activator Inhibitor-1

Plasminogen activator inhibitor-1 (PAI or PAI-1) inhibits tissue-type plasminogen activator (tPA or PLAT) and urinary kinase (uPA) and hereby fibrinolysis. Small case-control studies have pointed toward a role for PAI as a biomarker in lung disease [53,54,55]; however, this association has not yet been confirmed in cohort analyses [30,41,42,56], although some of the cohorts were based on RCTs. There are no studies analysing roles for uPa as a biomarker for lung injury, and similarly, any roles for tPa are currently occult [53].

### 3.6. Angiopoietin-2

Ang-2, also known as AGPT2, is expressed predominantly by the activated endothelium, and though its expression is low in quiescent mature vessels, it is strongly increased in inflammatory settings [57]. Higher levels of plasma Ang-2 seems to predict pulmonary affection in cohort studies in critically ill patients with various underlying courses: *N* = 230 in an emergency department [24], *N* = 439 with severe trauma [58], *N* = 84 who had undergone cardiac surgery [59], and *N* = 50 with septic shock and mechanical ventilation [60]. Ang-2 also seems to predict severity and mortality in patients with pulmonary damage. It predicted the severity of ARDS in 101 critically ill patients [61], and it predicted mortality in patients with ARDS in a cohort-based on the FACTT RCT: *N* = 252 on mechanical ventilation [62], *N* = 63 after surgical intervention [63], and *N* = 41 with sepsis [64] in addition to ARDS. However, there is also a cohort of 53 patients with ARDS in which Ang-2 was not able to predict the severity of illness or 28-day mortality [45], even though this study included 28 patients who died of ARDS. Hence, Ang-2 seems a promising biomarker for the development of ARDS as well as evaluation of severity and mortality.

### 3.7. Von Willebrand Factor

The von Willebrand factor (vWF) is involved in normal blood coagulation; however, it has also been proposed to be associated with inflammation. vWF does not predict the development of ARDS [23,24,65]. Similarly, vWF did not predict the development of multiple-organ failure or mortality in 100 patients with ARDS [30]; however, elevated plasma VWF levels did predict both the severity and mortality in 559 patients with ARDS [66]. Hence, vWF probably does not have a potential as a biomarker for ARDS; however, it may be a predictor for mortality in some subsets of patients with ARDS.

## 4. Treatment Response Biomarkers

### 4.1. Lung Inflammation Biomarkers

Previously, chronic obstructive pulmonary disease (COPD) exacerbation was thought to be triggered mainly by neutrophil-mediated inflammation, whereas eosinophilic inflammation was considered more characteristic of asthma. However, a subset of 20–40% of COPD patients display eosinophilic airway inflammation, even when those who potentially have asthma are excluded [67]. Eosinophils are important inflammatory and immune effector cells. Generally, eosinophils lie dormant in the blood, but upon exposure to proinflammatory cytokines (e.g., interleukin [IL]-3, IL-6, or granulocyte-macrophage colony-stimulating factor), they become activated and accumulate in inflamed tissue [68]. The presence of eosinophils in the lung is indicative of an abnormal inflammatory reaction [69]. Typically, eosinophils are quantified as a percentage of the total number of leukocytes or as the number of cells per µL, and these measurement methods agree in practice [67,70].

Blood eosinophil counts are often used as a proxy for eosinophilic lung inflammation because they correspond to sputum eosinophil counts [68]. Eosinophilic inflammation can occur when COPD is stable or during exacerbations [67]. Many studies have shown that blood eosinophil counts and relevant clinical COPD outcomes are linked. In addition, most of these studies found that an increased number of eosinophils in the blood (i.e., an eosinophilic COPD phenotype) was linked to poorer clinical outcomes such as an increased risk of hospital readmission, longer hospital stays, and future exacerbations [71,72,73,74,75]. In contrast to patients with stable COPD, those with acute exacerbations are usually treated with orally administered systemic corticosteroids such as prednisolone [76]. Systemic corticosteroids can promote recovery from symptoms but do not alleviate long-term declines in lung function, prevent future exacerbations after the first month, decrease the duration of intensive care treatment, or reduce mortality rates [77]. The criteria used to define COPD exacerbations (i.e., acute deterioration in respiratory symptoms that necessitates additional therapy) are broad and may encourage the overuse of corticosteroids [78]. In addition, the overuse of systemic corticosteroids may increase the risk of diabetes, osteoporotic fractures, cataracts, infections [79], and venous thromboembolism [80,81]. One possible strategy for limiting the use of systemic corticosteroids is to use blood eosinophil counts to guide corticosteroid treatment. An RCT that used blood eosinophils to categorise patients as having eosinophilic or non-eosinophilic exacerbations showed that treating non-eosinophilic exacerbations with placebo was not inferior to treating them with systemic corticosteroids. In particular, these researchers showed that there was a reduction of 49% in the total corticosteroid prescription for the eosinophil-guided group (*p* < 0.001) [82]. In addition, other studies have demonstrated that COPD patients with high blood eosinophil counts exhibit a better response to treatment with corticosteroids than do patients with low blood eosinophil counts [83,84,85]. Furthermore, the randomised non-inferiority CORTICO-COP trial was able to show that daily eosinophil counts could be used to guide treatment that reduced corticosteroid usage in hospitalised patients with COPD exacerbations. A reduction in median corticosteroid treatment duration from 5 days to 2 days was observed, with approximately two-thirds of the eosinophil-guided treatment patients taking no corticosteroids on any given day throughout the study, except on day 1. The treatment algorithm did not affect the number of days patients were alive and out of hospital within 14 days after recruitment or the 30-day all-cause mortality rate. In addition, among those patients with pre-existing diabetes in the eosinophil-guided corticosteroid group, fewer had hyperglycaemia or deteriorating of diabetes [86]. These analyses suggest that blood eosinophil count may be used as a biomarker to guide corticosteroid therapy for patients with COPD exacerbations and to decrease unnecessary exposure to systemic corticosteroids.

#### 4.1.1. Interleukin-1β

IL-1β, also known as leukocytic pyrogen, leukocytic endogenous mediator, mononuclear cell factor, and lymphocyte activating factor, is an important mediator of inflammation, but only a few studies have analysed its role as a biomarker for ARDS. A case-control study did not find different plasma levels of IL-1β in patients with ARDS, patients with severe pneumonia and healthy controls [87]; however, in a cohort study, elevated IL-1β levels predicted sepsis and mortality in 43 patients with ARDS [88]. Hence, the role for IL-1β as a biomarker is currently unclear.

#### 4.1.2. Interleukin-6

IL-6, also known as hepatocyte growth factor, B-Cell Stimulatory Factor 2, and Interferon-β-2, is a pro-inflammatory cytokine, which is induced by infections and tissue injury associated with any inflammation, such as increased level of Global Initiative for Chronic Obstructive Lung Disease severity in patients with COPD [89] and exacerbations of idiopathic pulmonary fibrosis [17]. Although IL-6 is not specific to pulmonary tissue, cohort and case-control studies have pointed toward a role for IL-6 in pulmonary inflammation and injury: IL-6 predicted the development of ARDS in 129 patients with severe sepsis [23] and 48 patients in an ICU [90]. IL-6 was associated with ARDS in case-control studies of patients with severe sepsis [12] and in patients with severe pneumonia [87,91].

IL-6 has also been studied for its ability to predict the severity of ARDS, including mortality. It predicted the severity of ARDS in 101 critically ill patients with new-onset fever [61], and it predicted sepsis and ICU mortality in 43 patients with ARDS [88], as well as the severity of ARDS and 28-day mortality in an analysis of two cohorts from RCTs (APC study with 75 patients and the ALVEOLI study with 259 patients) [41]. However, there are also a couple of studies that have not been able to show an association between IL-6 plasma levels and severity in patients with pulmonary injury. In 50 patients with early ARDS, there was no association between IL-6 by multivariate analysis, despite there being 21 non-survivors in the study [42]. Similarly, IL-6 did not predict mortality in 252 mechanically ventilated subjects with ARDS [62]. Hence, though IL-6 seems elevated in many patients with many kinds of lung injury, it may not always predict mortality in patients with lung injury.

#### 4.1.3. Interleukin-8

IL-8, also known as C-X- Motif Chemokine Ligand 8, anionic neutrophil-activating peptide, monocyte-derived neutrophil chemotactic factor lung, neutrophil chemotactant factor, and neutrophil-activating factor, is a chemokine produced by macrophages that induces the recruitment of especially neutrophils into a target tissue. Elevated IL-8 levels seem to predict the development of ARDS. In cohort studies, IL-8 predicted the development of ARDS in 230 critically ill patients [24], 172 patients with sepsis or septic shock [65], 129 patients with severe sepsis [23], and in 48 patients in intensive care [90]. A similar correlation was seen in case-control studies [12,54,92].

In patients with lung injury, IL-8 may also to some degree predict the severity of disease and mortality; however, studies on severity and mortality do not yet draw a clear picture of its potential, as they are few and included relatively few patients. In cohort studies, IL-8 predicted fewer days without mechanical ventilation and 28-day mortality in 259 critically ill patients with ARDS [62], and both the development of sepsis and ICU mortality in 43 patients with ARDS [88], and mortality in a small cohort study of 30 patients with ARDS [93]. However, there was also a cohort study of 100 patients with fully developed ARDS, in which IL-8 could not predict mortality, but it did predict multiple organ failure [30]. In a small case-control study (*N* = 24 patients with ARDS), IL-8 was also correlated to mortality [94].

#### 4.1.4. Interleukin-10

Il-10, also known as Cytokine Synthesis Inhibitory Factor, is an anti-inflammatory cytokine that inhibits the synthesis of IL-1 and Tumor Necrosis Factor-α (TNF-α). A small case-control study showed a role for decreased IL-10 in bronchoalveolar lavage fluid; however, subsequently, two cohort studies [95] of 861 and 107 patients with ARDS have demonstrated no ability of plasma IL-10 as a biomarker in lung injury [96,97].

### 4.2. Tumour Necrosis Factor-α

In two case-control studies, plasma levels of TNF-α were associated to ARDS compared to controls in a trauma intensive care setting [92] and to ARDS compared to patients with severe pneumonia without ARDS [87], and in a cohort study of 43 patients with ARDS, TNF-α predicted the development of sepsis and mortality [88]. A combination of clinical predictors with a combination of seven biomarkers (TNF-α, s-RAGE, collagen deposition (PCPIII), brain natriuretic peptide, Ang-2, IL-10, and IL-8) performed well for differentiating ARDS cases from controls with an area under the ROC curve of 0.86 [92]. Hence, TNF-α, IL-8, and IL-10 may be a potential biomarker for ARDS, but more studies need to be conducted in this area.

### 4.3. Lung Infection Biomarkers

The observational studies conducted to date have not determined whether procalcitonin (PCT)-guided antibiotic treatment initiation or intensification increases the likelihood of survival in patients who are critically ill with sepsis. The Procalcitonin And Survival Study explored whether PCT-guided initiation or intensification of treatment with antibiotics and other antimicrobial measures could increase the probability that patients would survive by substantially decreasing the time until adequate antibiotics were administered [98]. This RCT study (*N* = 1200) involved nine intensive care units across Denmark. In the active treatment group, an increase in PCT level led to a wider range of antibiotics being used for treatment according to a specified algorithm and elicited additional culture sampling and radiological imaging of suspected infected foci [99]. Despite a high level of adherence to the antimicrobial intervention algorithm (82%), the study intervention did not increase the likelihood of patient survival. The explanation for this result may be that there is a “neutralising” effect, involving harm from antibiotics counteracting any benefits from better timing of their administration. Notably, several harmful effects were reported from the “high-intensive” antibiotic strategy in the PCT-guided arm of the trial: an increase in the risk of renal failure that was attributed to piperacillin [100], a ciprofloxacin-associated increase in the risk of invasive fungal infection [101] and haematological side effects [102]. Most of the patients who had been classified as high risk (i.e., those with severe sepsis or septic shock) were already being given broad-spectrum antibiotics. Consequently, there was little scope for expanding this spectrum in a way that would likely alter prognoses. Therefore, increasing PCT levels in patients who are critically ill with sepsis should not necessarily lead to more intensive antimicrobial treatments.

### 4.4. Decreasing the Use of Antibiotics in Patients with Acute Respiratory Infections by Monitoring PCT Levels

According to the World Health Organisation, “antibiotic resistance is one of the biggest threats to global health, food security, and development today” and “antibiotic resistance occurs naturally, but misuse of antibiotics in humans and animals is accelerating the process”. As a result, many international initiatives have sought to reduce unnecessary antibiotic use and alleviate the problem of antibiotic resistance. Continuous assessment of PCT levels has been investigated as a guide to terminating antibiotic treatment of patients with acute respiratory tract infections in various settings including primary care, emergency care [103], patients with bacteraemia [104], and intensive care [105]. In all of these settings, when an intervention protocol could be implemented, and serial measurements recorded, antibiotic treatment durations could be decreased substantially. The results from these studies and similar research have been collated in a systematic review with sufficient power to explore mortality rates and the side effects of antibiotics [106]. The PCT-guided protocols led to a reduction in the defined side effects of antibiotics from 22% to 16% and, surprisingly, mortality rates also decreased; conversely, in a recent trial involving patients with acute respiratory tract infections who were recruited before hospital admission, decreasing antibiotic treatment durations resulted in no apparent benefit. In this trial, the decision to admit patients to hospital (49.7% of those assessed) and the duration of treatment with antibiotics were both determined by PCT levels. One reason why reducing the duration of antibiotic therapy in the trial produced no clear benefit may be that serial measurements were only performed for half of the patients (i.e., those who were admitted to hospital). In addition, protocol adherence among patients who had PCT levels that were low enough to allow them to discontinue antibiotic treatment was only 30–45%. If the results of the PCT measurements do not lead to appropriate changes in treatment, the strategy will not be effective.

### 4.5. C-Reactive Protein

The role of C-reactive protein (CRP) as a biomarker for ARDS is currently quite unclear. Higher CRP was related to the severity of ARDS in 101 critically ill patients in an ICU setting [107], but in another cohort study, lower CRP predicted organ failure, the need for mechanical ventilation, and 60-day mortality in 177 patients with ARDS [108]. In a third cohort study, CRP could not predict severity of ARDS nor mortality [50]. Hence, the role for CRP as a biomarker for ARDS remains uncertain.

### 4.6. White Blood Cells

White blood cells (WBC) and especially neutrophils may also be proposed as possible biomarkers for lung injury; however, despite the vast abundance of patients with ARDS who must have had these factors tested daily over the last 50 years, published studies in this area are few. So far, studies have shown that neither WBC nor neutrophils predicted the severity or mortality in patients with ARDS [50]. Monocytes might predict ARDS based on a study of a cohort from the LIPS-A RCT [109], regulatory T-cells might be associated with ARDS [110], mononuclear cells in patients with ARDS might have a greater potential for colony formation [111], and a high neutrophil-to-lymphocyte ratio might predict mortality in critically ill patients with ARDS [112] (Table 2). Hence even the role of WBC as biomarkers for ARDS remains unenlightened.

## 5. Conclusions

The identification of definitive biomarkers capable of diagnosing ARDS, predicting prognoses, and monitoring responses to disease treatment would present new opportunities for progress in this research field. The discovery and validation of a biomarker or set of biomarkers would help identify ARDS patients, quantify the severity of lung injuries, and guide treatment strategies. Many potential biomarkers have been investigated, but a single biomarker that can reliably diagnose ARDS specifically has not yet been found. Since the pathophysiology of ARDS is complex and heterogeneous, current research suggests that combinations of biomarkers that reflect different aspects of ARDS (such as epithelial and endothelial injury, inflammation or infection) are more likely to be use in a clinical context. Indeed, the best approach will probably combine clinical predictors with several biomarkers as has been suggested and tested with varying degrees of success in quite a few studies by now, including cohorts based on several RCTs [8,9,10,65,92,113,114]. However, none of these candidates have been used clinically in patients with ARDS. Future studies should determine the potential for each candidate discussed here. This will lead to improved diagnoses and treatments strategies for patients with ARDS.

## Figures and Tables

**Table 1:** Berlin criteria for acute respiratory distress syndrome (ARDS) [3]. PEEP: positive end-expiratory pressure, PaO_2_: arterial oxygen tension, FiO_2_: inspiratory oxygen fraction, CT: computed tomography.

	BERLIN CRITERIA
TIMING	Within 1 week of a known clinical insult or new or worsening respiratory symptoms
OXYGENATION	Mild: PaO_2_/FiO_2_ > 200 mmHg but ≤ 300 mmHg Moderate: PaO_2_/FiO_2_ > 100 mmHg but ≤ 200 mmHg Severe: PaO_2_/FiO_2_ ≤ 100 mmHg
PEEP REQUIREMENT	Minimum 5 cm H_2_O PEEP required by invasive mechanical ventilation (noninvasive acceptable for mild ARDS)
CHEST IMAGING	Bilateral opacities not fully explained by effusions, lobar/lung collapse or nodules by chest radiograph or CT
ORIGIN OF OEDEMA	Respiratory failure not fully explained by cardiac failure or fluid overload (need objective assessment, such as echocardiography, to exclude hydrostatic oedema if no risk factor present)

**Table 2:** Biomarkers for acute respiratory distress syndrome (ARDS).

Pathophysiological Entity for Biomarker	Biomarker	Clinical Use Potential
*Alveolar and bronchiolar damage*	Surfactant Protein D (SP-D)	Diagnosis and risk stratification of lung diseasespecifically ARDS [11,12]
	Krebs von den Lungen-6 (KL-6)	Indication of alveolar injury in ARDS patients and prognostic biomarker [13,14,15,16,20]
*Endothelial Damage*	Gelsolin, actin scavenging protein	Prediction of respiratory outcome in mechanically ventilated patients [37]
	Syndecan-A	Prognosis for pre-existing respiratory failure [40]
	Angiopoetin-2 (Ang-2)	Prediction severity and mortality in ARDS [61,62]
	Soluble FMS-like tyrosin kinase 1	Prediction of mortality [38]
	Soluble VEGF-receptor	Prediction of mortality [38]
	Von Willebrand factor (vWF)	Prediction of mortality in some ARDS patients [66]
	Thrombomodulin (TM)	Possible indicator of mortality [39,40] Prediction of severity and complications in ARDS patients [41]
	Protein C	Prediction of ARDS mortality [43,44]
	Endocan	Unclear [45,46,47,48,49,50,51,52]
	Plasminogen activator inhibitor- 1 (PAI)	Possible usefulness as biomarker in ARDS [30,41,42,53,54,55,56]
	sRAGE	Prediction of ARDS mortality [7,26] Diagnosis of ARDS [12,25,35]
*Treatment response*		
*Lung inflammation*	Blood eosinophil count	Guidance and reduction of corticosteroid treatment and prediction of response [83,84,85,86]
	IL-1β	Possible prediction of sepsis and mortality in ARDS [88]
	IL-6	Prediction of ARDS development, severity and mortality [12,23,41,87,88,90,91]
	IL-8	Prediction of ARDS development [23,24,65,90] Prediction of severity and mortality in patients with lung injury [62,88,93]
	IL-10	Unclear role in ARDS prediction [95,96,97]
	TNF-α	Associated with ARDS [87,92] Potential prediction of sepsis and mortality in ARDS [88]
*Antibiotic reduction*	Procalcitonin (PCT)	Reduction in use of antibiotics [106]
*Lung infection*	C-reactive protein (CRP)	Possible role in predicting ARDS severity [107,108]
	White blood cells (WBC)MonocytesRegulatory T-cellsNeutrophil-to-lymphocyte ratio	No prediction of severity or mortality in ARDS [50] Prediction of ARDS [109] Associated with ARDS [110] Prediction of ARDS mortality [112]

## Data Availability

No new data were created or analyzed in this study. Data sharing is not applicable to this article.
